# Persistent DNA methylation changes associated with prenatal mercury exposure and cognitive performance during childhood

**DOI:** 10.1038/s41598-017-00384-5

**Published:** 2017-03-21

**Authors:** Andres Cardenas, Sheryl L. Rifas-Shiman, Golareh Agha, Marie-France Hivert, Augusto A. Litonjua, Dawn L. DeMeo, Xihong Lin, Chitra J. Amarasiriwardena, Emily Oken, Matthew W. Gillman, Andrea A. Baccarelli

**Affiliations:** 1 Department of Environmental Health, Harvard T.H. Chan School of Public Health, Boston, MA USA; 2 Obesity Prevention Program, Department of Population Medicine, Harvard Medical School and Harvard Pilgrim Health Care Institute, Boston, MA USA; 3 Channing Division of Network Medicine, Department of Medicine, Brigham and Women’s Hospital, Boston, MA USA; 4 Department of Biostatistics, Harvard T.H. Chan School of Public Health, Boston, MA USA; 5 Department of Preventive Medicine, Icahn School of Medicine at Mount Sinai, New York, NY USA

## Abstract

Prenatal exposure to mercury, a known neurotoxic metal, is associated with lower cognitive performance during childhood. Disruption of fetal epigenetic programming could explain mercury’s neurodevelopmental effects. We screened for epigenome-wide methylation differences associated with maternal prenatal blood mercury levels in 321 cord blood DNA samples and examined the persistence of these alterations during early (n = 75; 2.9–4.9 years) and mid-childhood (n = 291; 6.7–10.5 years). Among males, prenatal mercury levels were associated with lower regional cord blood DNA methylation at the Paraoxonase 1 gene (*PON1*) that persisted in early childhood and was attenuated in mid-childhood blood. Cord blood methylation at the *PON1* locus predicted lower cognitive test scores measured during early childhood. Methylation at the *PON1* locus was associated with PON1 expression in an independent set of cord blood samples. The observed persistent epigenetic disruption of the *PON1* gene may modulate mercury toxicity in humans and might serve as a biomarker of exposure and disease susceptibility.

## Introduction

Mercury (Hg) is a ubiquitous worldwide environmental contaminant that can persist in the environment and bioaccumulates as methylmercury (MeHg) in the food chain. Elemental mercury is a natural metal that is primarily introduced in the air, water and food from coal burning power plants, artisanal mining activities and industrial applications^1^. Since the industrial revolution, mercury levels in surface water have tripled^2^. For the general public, the consumption of fish or seafood containing MeHg is the major source of exposure^3^.

MeHg crosses the placenta and the blood-brain barrier and accumulates in fetal tissues resulting in fetal blood concentrations that typically exceed maternal levels^4^. Prenatal effects of acute MeHg exposure were first documented in 1956, when industrial activity heavily polluted Japan’s Minamata bay causing coastal residents to consume highly contaminated seafood. Some infants born during this period suffered from severe neurological disabilities and fetal abnormalities^5^. Recent prospective epidemiological studies have shown that even moderate or low-level prenatal exposure to mercury *in utero* typical of regular fish consumption may be associated with lower cognitive test scores in children^6–10^. However, the dose-response relationship, mechanism of action and potential effect modifiers such as gender and timing of exposure have not been fully evaluated^11^.

The specific mechanisms of neurotoxicity and cognitive disruption associated with prenatal mercury exposure remain poorly characterized in humans. Nonetheless, the period of fetal development has been shown to be sensitive to prenatal exposure, perhaps in part because of the dramatic DNA methylation changes and epigenomic remodeling that takes place early during embryogenesis, giving rise to cells and tissues with specific DNA methylation patterns^12^. The ability of mercury to cross the placenta and blood-brain barrier during development makes it a candidate toxicant for the disruption of fetal programming events that could propagate through different germ layers during embryogenesis.

Although several studies have characterized DNA methylation changes in cord blood relative to prenatal environmental exposures, very few studies have evaluated the persistence of these changes into childhood^13^. Evaluating the persistence of epigenetic modifications is critical as DNA methylation is a dynamic process that can drift with age and other stochastic processes^14^. To date, three epigenome-wide association studies have been conducted identifying unique genomic regions as well as individual CpG methylation disruption in cord blood^15, 16^ and placenta^17^ of newborns prenatally exposed to mercury. However, these studies were limited in sample size (ranging from 41 to 141 samples) and did not evaluate whether the observed epigenetic alterations were persistent, sex-specific or related to cognitive performance in childhood. Epigenetic responses to environmental conditions can be sex specific in terms of perception of exposure and subsequent response, particularly for neurodevelopmental disorders^18, 19^. Furthermore, gender differences in MeHg neurotoxicity have been documented in epidemiological and animal models^20, 21^. Therefore, it is crucial to evaluate sex-specific epigenetic alterations in response to environmental conditions.

In the present study, we examined associations of prenatal mercury exposure with epigenome wide DNA methylation in cord blood, and evaluated if these epigenetic modifications persist in blood through early and mid-childhood. We also evaluated sex-specific differences in response to prenatal mercury exposure. We hypothesized that persistent DNA methylation modifications in cord blood related to prenatal mercury exposure would also be associated with cognitive test scores measured in early childhood.

## Results

**Figure 1:**
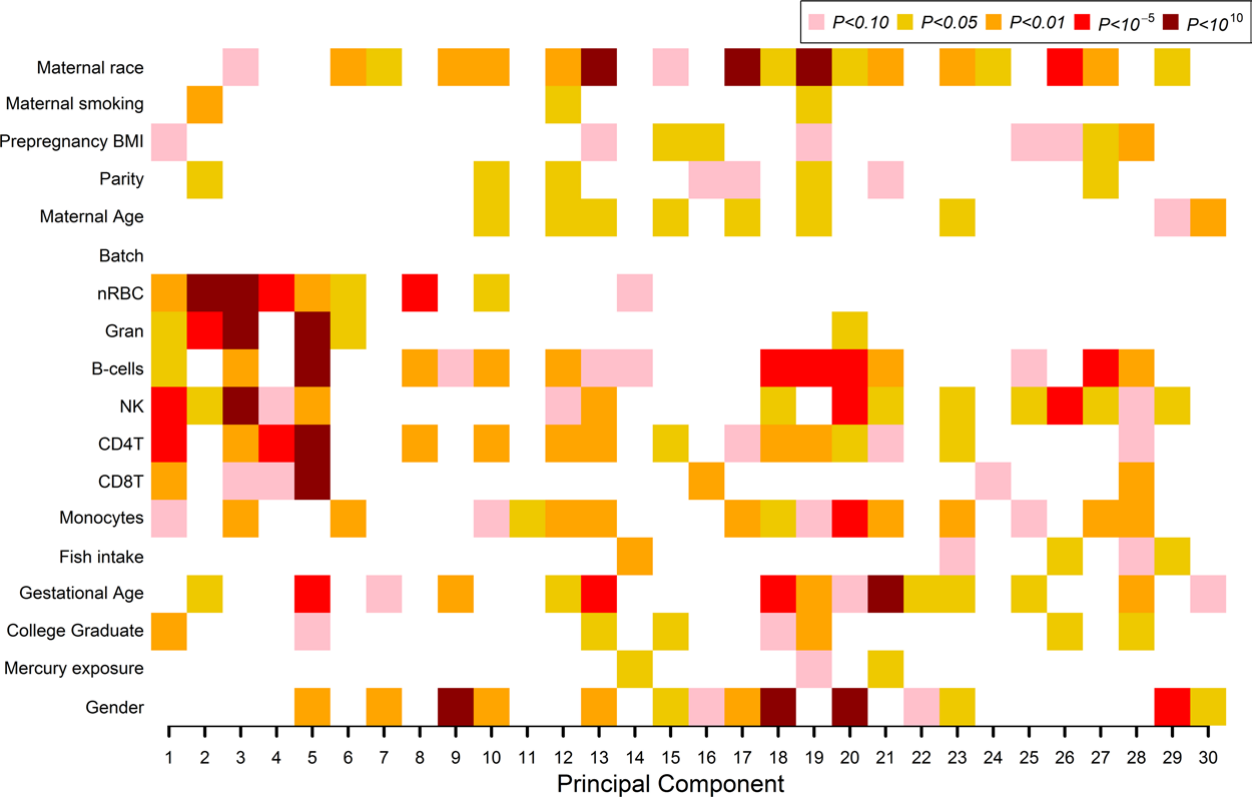
Principal component regression analysis: univariate association *P*-values between covariates of interest and the top 30 principal components that explain 51.4% of the variance for the entire DNA methylation data in cord blood.

A total of 321 mother-child pairs had available data on both maternal prenatal mercury exposure and cord blood DNA methylation. During early childhood (range: 2.9 to 4.9 years), 75 participants had whole blood samples available for analysis and 291 participants had samples available for mid-childhood analyses (range: 6.7 to 10.5 years), Table 1. Mean maternal red blood cell mercury (RBC-Hg) concentration measured during the second trimester of pregnancy was 3.8 ng/g (SD = 3.1) and mean maternal fish intake during pregnancy was 1.5 servings per week (SD = 1.2). Prenatal mercury concentration in maternal erythrocytes was right skewed and so we log_2_-transformed it for analyses to approximate a log-normal distribution, Supplementary Fig. S1. Univariate associations among the main 30 principal components of DNA methylation data, explaining 51.4% of the variance, showed strong associations with estimated cell type composition and moderate associations with phenotype characteristics after the effective removal of technical batch effects, Fig. 1.

**Table 1:** Characteristics of mothers and children in the Project Viva eligible for analysis at birth (cord blood) and during early (2.9–4.9 years) and mid-childhood (6.7–10.5 years).

Study characteristic	Cord Blood	Early childhood	Mid-childhood
	N = 321	N = 75	N = 291
**Maternal characteristics**	**Mean (SD) or n (%)**		
Pre-pregnancy BMI (kg/m^2^)	24.2 (4.9)	25.6 (6.0)	24.4 (4.9)
Age at enrollment (years)	31.9 (5.0)	32.0 (4.6)	32.2 (5.4)
Nulliparous			
No	167 (52%)	41 (54.7%)	157 (54%)
Yes	154 (48%)	34 (45.3%)	134 (46%)
College graduate			
No	106 (33%)	21 (28%)	89 (30.6%)
Yes	215 (67%)	54 (72%)	202 (69.4%)
Smoking status			
Never	216 (67.3%)	44 (58.7%)	209 (71.8%)
Former	67 (20.9%)	18 (24.0%)	53 (18.2%)
During pregnancy	38 (11.8%)	13 (17.3%)	29 (10%)
Any alcohol during pregnancy			
No	87 (27.1%)	21 (28.0%)	99 (34%)
Yes	234 (72.8%)	54 (72.0%)	192 (66%)
Race/ethnicity			
White	248 (77.3%)	61 (81.3%)	206 (70.8%)
Black	28 (8.7%)	5 (6.7%)	45 (15.5%)
Hispanic	21 (6.5%)	2 (2.7%)	14 (4.8%)
Other	24 (7.5%)	7 (9.3%)	26 (8.9%)
PPVT scores	105.7 (14.3	107.0 (13.0)	105.3 (16)
2^nd^ trim RBC-Hg (ng/g)	3.8 (3.1)	4.2 (3.7)	4.0 (3.2)
2^nd^ trim fish intake (servings/week)	1.5 (1.2)	1.3 (1.1)	1.7 (1.5)
**Child characteristics**	**Mean (SD) or n (%)**		
Sex			
Male	160 (49.8%)	37 (49.3)	149 (51.2%)
Female	161 (50.1%)	38 (50.7)	142 (48.8%)
Age at sample collection			
Birth, (gestational age in weeks)	39.7 (1.6)	—	—
Early childhood (2.9 to 4.9 years)	—	3.4 (0.5)	—
Mid-childhood (6.7 to 10.5 years)	—	—	7.9 (0.8)
Race/ethnicity			
White	234 (72.9%)	58 (77.3%)	194 (66.7%)
Black	31 (9.7%)	5 (6.7%)	46 (15.8%)
Hispanic	14 (4.4%)	1 (1.3%)	11 (3.8%)
Other	42 (13.1%)	11 (14.7%)	40 (13.7%)
Gestational age at birth (weeks)	39.7 (1.6)	39.6 (1.6)	39.6 (1.6)
PPVT score early childhood	—	103.8 (12.9)	—
WRAVMA score early childhood	—	103.6 (9.7)	—

### Regional DNA Methylation Analysis

**Figure 2:**
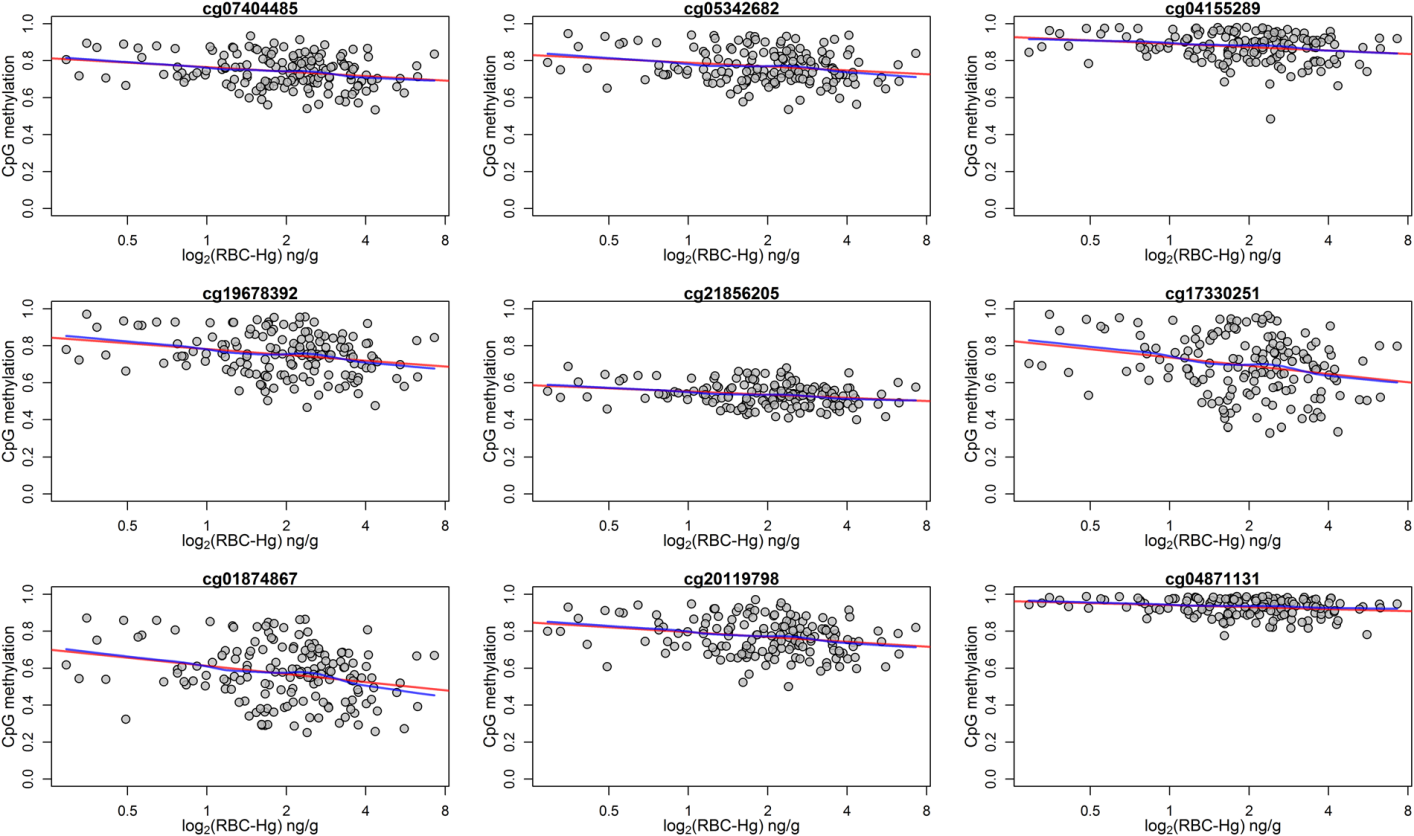
Scatterplots for the associations between individual cord blood CpG methylation in the DMR of the *PON1* gene and log_2_-transformed prenatal mercury exposure: red (fitted simple linear regression line) blue (locally weighted scatterplot smoothing).

**Figure 3:**
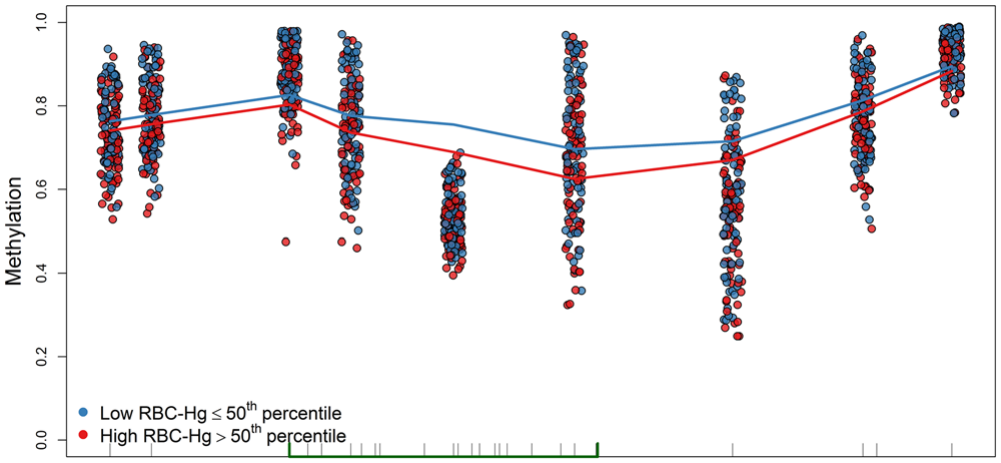
Cord blood CpG methylation distribution and fitted locally weighted scatterplot smoothing (LOESS) lines in the DMR found in *PON1* (9 CpGs) with low (blue) and high (red) prenatal mercury exposure among males. Rug plot represents the estimated CpG density in the region while the green area denotes a CpG Island.

**Figure 4:**
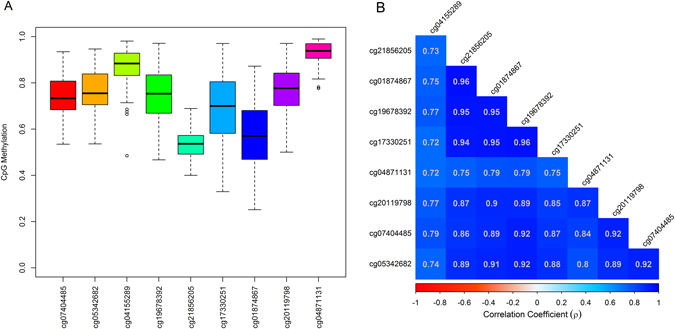
CpG sites in the differentially methylated region of *PON1* gene associated with prenatal mercury exposure: (**A**) Boxplots for the distribution of DNA methylation levels at each CpG site and (**B**) correlations among all nine CpGs in the *PON1* DMR.

To identify Differentially Methylated Regions (DMRs) in the genome relative to prenatal mercury exposure, we implemented a regional based approach that is agnostic to both genomic annotation and direction of individual CpG associations. After stratifying on sex, a DMR covering 9 CpGs of chromosome 7 in the Paraoxonase 1 gene (*PON1*) was hypomethylated by prenatal mercury exposure among boys (genomic coordinates: chr7:94,953,653–94,954,202; FDR < 0.05), Fig. 2. We did not identify any DMRs in girls, or in the overall population. Among males, the multivariate adjusted magnitude of association for each individual CpG site in the *PON1* DMR ranged from a 1% to a 3.8% decrease in individual cord blood CpG methylation per doubling in maternal RBC-Hg concentrations, Table 2. Overall, mean cord methylation levels of all nine CpGs located in the *PON1* locus were inversely associated with maternal RBC-Hg concentrations (β = −2.4%, 95% CI: −3.8, −1.0; *P* = 7.5 × 10^−4^), Table 2. Scatter plots with locally weighted smoothing lines by median prenatal mercury concentrations levels of the *PON1* DMR are shown in Fig. 3. Individual cord blood methylation levels for the 9 CpGs in this DMR ranged from 25.2% to 98.9% (Fig. 4A) and they were strongly positively correlated with each other (ρ-range: 0.72 to 0.96), Fig. 4B.

**Table 2:** Adjusted percent change in blood DNA methylation of males per doubling in prenatal mercury exposure for the differentially methylated region in the *PON1* gene at birth (cord blood) and persistence during early and mid-childhood.

*PON1* DMR*-*CpGs		Cord Blood (N = 160)		Early childhood (N = 37)		Mid-childhood (N = 149)	
CpG ID	Gene Region	β-Coefficient (95% CI)	*P*	β-Coefficient (95% CI)	*P*	^‡^β-Coefficient (95% CI)	*P*
cg07404485	Body	−2.2% (−3.5, −0.9)	7.2 × 10^−4^	−4.2% (−7.8, −0.7)	0.02	−1.1% (−2.5, 0.3)	0.11
cg05342682	Body	−1.8% (−3.2, −0.5)	8.0 × 10^−3^	−5.5% (−9.7, −1.3)	0.01	−1.2% (−2.5, −0.01)	0.04
cg04155289	1stExon	−1.7% (−2.8, −0.6)	3.3 × 10^−3^	−3.4% (−9.4, 2.5)	0.26	−0.6% (−1.6, 0.4)	0.18
cg19678392	1stExon; 5′UTR	−2.9% (−4.6, −1.2)	8.5 × 10^−4^	−4.9% (−12.7, 2.8)	0.21	−1.8% (−3.3, −0.3)	0.02
cg21856205	1stExon; 5′UTR	−1.6% (−2.5, −0.6)	9.9 × 10^−4^	−3.1% (−5.5, −0.6)	0.01	−0.8% (−1.6, −0.01)	0.03
cg17330251	TSS200	−3.7% (−5.9, −1.4)	1.6 × 10^−3^	−6.7% (−16.6, 3.1)	0.18	−2.1% (−4.1, −0.1)	0.04
cg01874867	TSS200	−3.8% (−6.1, −1.6)	8.2 × 10^−4^	−6.5% (−12.8, −0.3)	0.04	−2.2% (−4.1, −0.3)	0.02
cg20119798	TSS1500	−2.7% (−3.9, −1.4)	2.5 × 10^−5^	−5.3% (−9.8, −0.7)	0.02	−1.1% (−2.7, 0.4)	0.15
cg04871131	TSS1500	−1.0% (−1.6, −0.5)	1.5 × 10^−4^	−1.9% (−4.1, 0.3)	0.09	−0.2% (−1.0, 0.5)	0.52
Mean DMR methylation		−2.4% (−3.8, −1.0)	7.5 × 10^−4^	−4.6% (−9.0, −0.1)	0.044	−1.2% (−2.5, 0.1)	0.06

To evaluate the persistence of association between prenatal mercury exposure and DNA methylation at the *PON1* locus found in cord blood, we tested associations between prenatal mercury exposure with DNA methylation at the *PON1* locus in blood collected in males at early and mid-childhood. In early childhood (n = 37), a doubling in prenatal mercury concentration was associated with a 4.6% decrease in mean methylation levels of the DMR in *PON1*, measured across the nine CpGs (β = −4.6%, 95% CI: −9.0, −0.1; *P* = 0.044). Investigating all 9 CpGs individually, the inverse adjusted association of prenatal mercury exposure reached statistical significance in early childhood among five CpGs: cg07404485 (β = −4.2%, 95% CI: −7.8, −0.7), cg05342682 (β = −5.5%, 95% CI: −9.7, −1.3), cg21856205 (β = −3.1%, 95% CI: −5.5, −0.6), cg01874867 (β = −6.5%, 95% CI: −12.8, −0.3) and cg20119798 (β = −5.3%, 95% CI: −9.8, −0.7). In mid-childhood (n = 149), mean methylation levels of the *PON1* DMR were marginally associated with prenatal mercury exposure for males (β = −1.2%, 95% CI: −2.5, 0.1; *P* = 0.06). However, five individual CpG sites within the *PON1* region were associated with maternal RBC-Hg concentrations: cg05342682 (β = −1.2%, 95% CI: −2.5, −0.01), cg19678392 (β = −1.8%, 95% CI: −3.3, −0.3), cg21856205 (β = −0.8%, 95% CI: −1.6, −0.01), cg17330251 (β = −2.1%, 95% CI: −4.1, −0.1) and cg01874867 (β = −2.2%, 95% CI: −4.1, −0.3). Persistence of the association for the male-specific DMR in *PON1* and prenatal RBC-Hg concentrations is summarized in Table 2.

### Overall CpG-by-CpG analysis in cord blood

**Figure 5:**
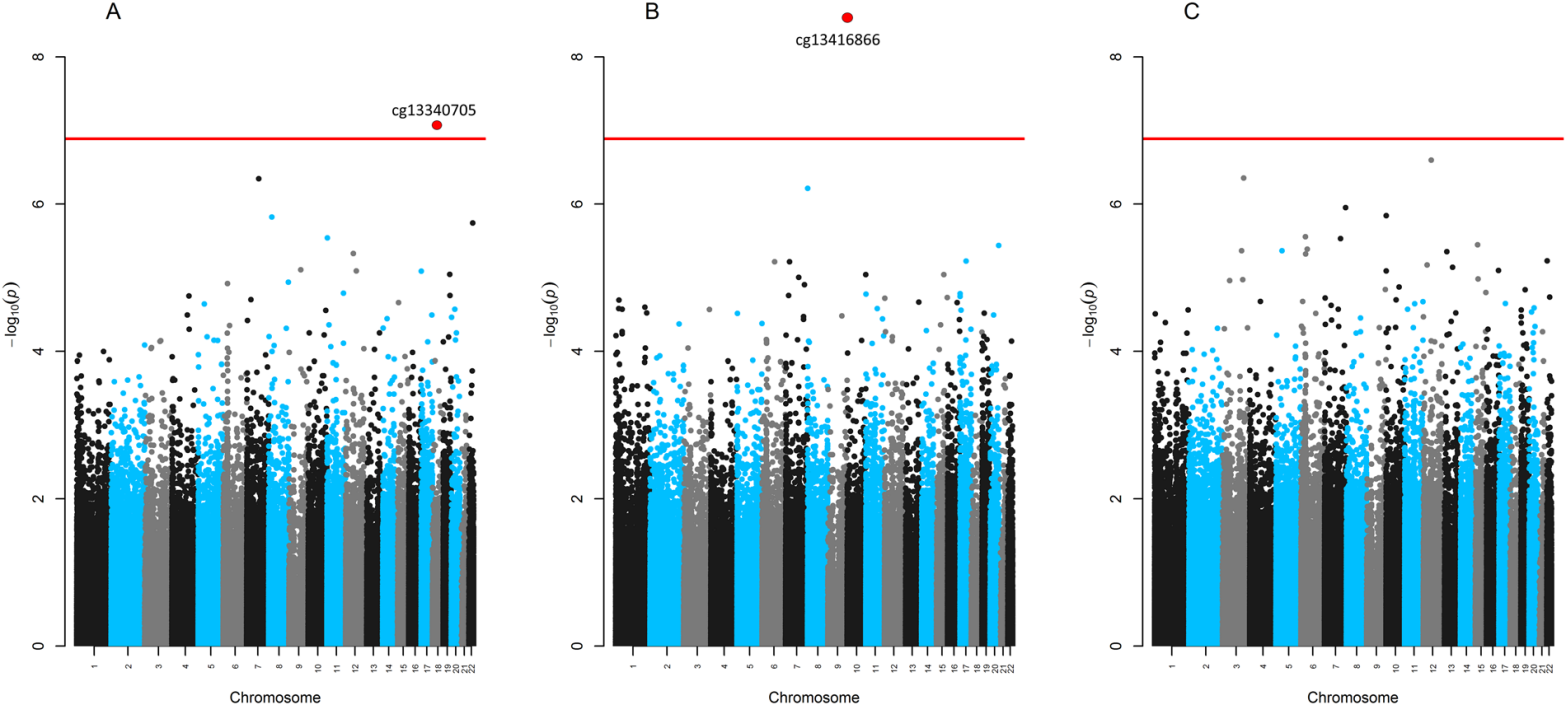
Manhattan plots for the epigenome-wide association of prenatal mercury exposure and individual CpG methylation levels: (**A**) Overall analysis (**B**) males-specific analyses and (**C)** female-specific analyses (red solid line represents the Bonferroni adjusted level of significance).

In adjusted robust linear regression models, one CpG was differentially methylated relative to prenatal mercury exposure after using a Bonferroni adjusted level of significance, Fig. 5A. Namely, for every doubling in maternal RBC-Hg concentrations a 0.3% increase in cord blood methylation was observed for cg13340705 annotated to the WW Domain Binding Protein 11 Pseudogene 1 (*WBP11P1*) located in chromosome 18 (β = 0.3%, 95% CI: 0.2, 0.5; *P* = 5.3 × 10^−7^). However, this association did not persist in early (β = −0.1%, 95% CI: −0.5, 0.3; *P* = 0.57) or mid-childhood (β = 0.03%, 95% CI:−0.2, 0.2; *P* = 0.81), Table 3.

**Table 3:** Adjusted percent change in blood DNA methylation per doubling in prenatal mercury exposure for individual loci found to be differentially methylated at birth (cord blood) in the CpG-by-CpG analysis and persistence of associations during early and mid-childhood.

Overall CpG-by-CpG analyses			Cord Blood (N = 321)		Early childhood (N = 75)		Mid-childhood (N = 291)	
CpG ID	Gene	CHR	β-Coefficient (95% CI)	*P*	β-Coefficient (95% CI)	*P*	β-Coefficient (95% CI)	*P*
cg13340705	*WBP11P1*	18	0.3% (0.2, 0.5)	5.3 × 10^−7^	−0.1% (−0.5, 0.3)	0.57	0.03% (−0.2, 0.2)	0.81
**Male-specific CpG-by-CpG analyses**			**Cord Blood (N = 160)**		**Early childhood (N = 37)**		**Mid-childhood (N = 149)**	
cg13416866	*TOR4A*	9	1.3% (0.8, 1.7)	3.5 × 10^−8^	0.6% (−0.7, 2.0)	0.34	0.72% (0.1, 1.4%)	0.031

### Sex-specific CpG-by-CpG analysis in cord blood

After stratifying by sex, one CpG in males (cg13416866) annotated to the Torsin Family 4, Member A gene (*TOR4A*) was hypermethylated relative to maternal RBC-Hg concentrations (β = 1.3%, 95% CI: 0.8, 1.7; *P* = 3.5 × 10^−8^), Fig. 5B. The effect estimate for the association between prenatal mercury exposure and methylation levels of this CpG measured during early childhood was similar in direction (β = 0.6%, 95% CI: −0.7, 2.0; *P* = 0.34) but not significant, likely due to sample size (N = 37). However, the association for this CpG was significant for methylation levels measured in blood at mid-childhood (β = 0.72%, 95% CI: 0.1, 1.4; *P* = 0.031), Table 3. No sites were found to be differentially methylated in cord blood for females in the CpG-by-CpG analyses, Fig. 5C.

### DNA Methylation and Gene Expression

**Figure 6:**
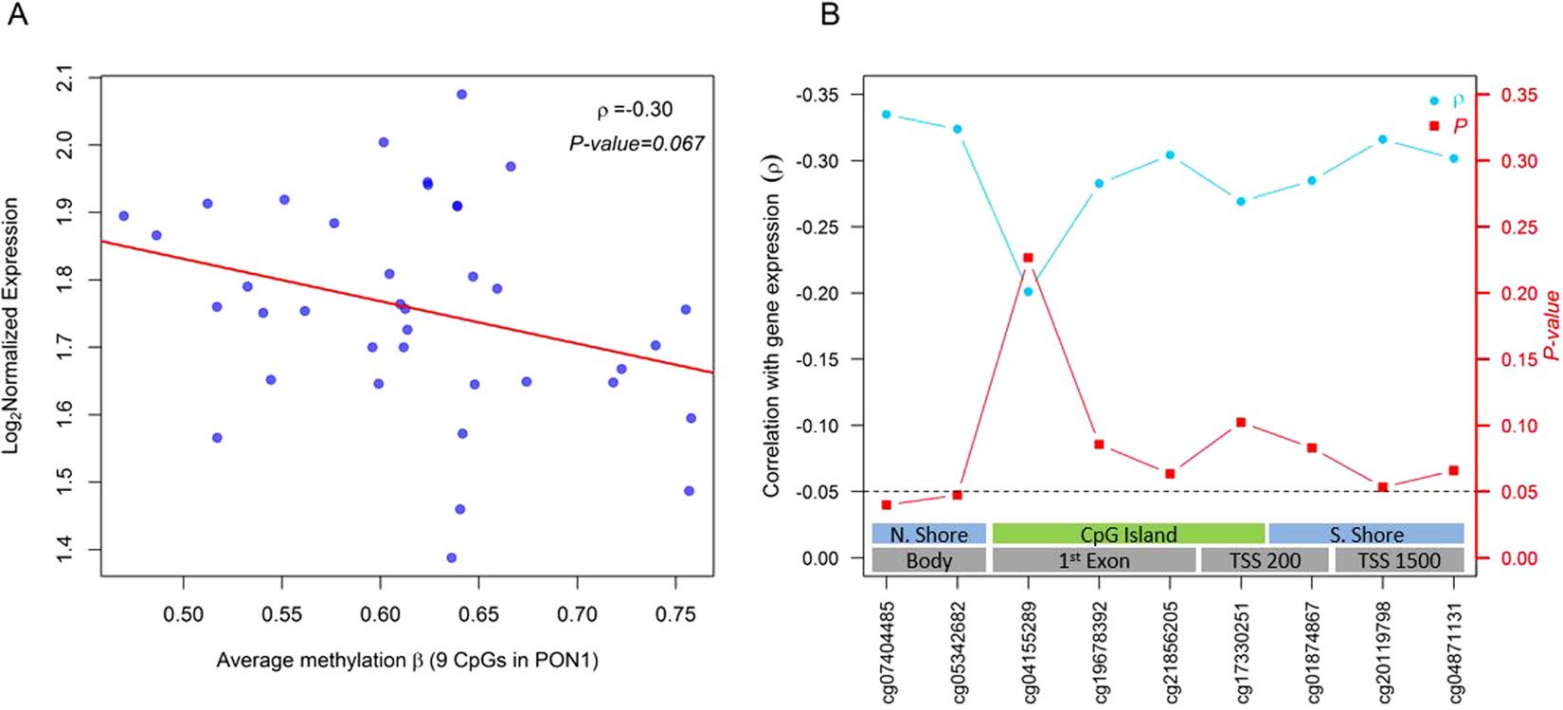
Association between DNA methylation and gene expression of the *PON1* gene at the DMR associated with prenatal mercury exposure from an independent birth cohort (N = 38): (**A**) scatter plot of mean methylation levels of the DMR in *PON1* and gene expression and (**B**) individual correlation coefficients and corresponding *P*-values for each individual CpG site in the DMR.

To evaluate the functional relevance of methylation changes, we examined whether DNA methylation of the *PON1* DMR and differentially methylated single CpGs were correlated with gene expression in cord blood from an independent birth cohort (Biomarkers of Exposure to Arsenic; N = 38)^22^. In this independent set of samples we observed a moderate negative correlation between *PON1* RNA expression and mean methylation levels of the nine CpGs in the DMR that approached statistical significance (ρ = −0.30, *P* = 0.06), Fig. 6A. Two CpGs in the *PON1* DMR located in the body of the gene and in the north shore region of a CpG island had the strongest association with expression: cg07404485 (ρ = −0.33, *P* = 0.04) and cg05342682 (ρ = −0.32, *P* = 0.04), respectively. Five other loci from the DMR reached marginal significance (*P* < 0.10), Fig. 6B. None of the CpGs found to be associated with prenatal mercury exposure in the CpG-by-CpG analyses correlated with gene expression of cord blood samples, Supplementary Table S1. The genomic tracks of the DMR in *PON1* and annotation for the gene expression array used, mRNA, known SNPs and CpGs are shown in Supplementary Fig. S2.

### DNA Methylation and Child Cognitive Performance

In males, mean methylation levels in cord blood for the *PON1* DMR were marginally associated with cognitive PPVT scores measured in early childhood (β = −2.6, 95% CI: −5.6, 0.3; *P* = 0.07). Within the *PON1* DMR, four individual CpGs were significantly associated with cognitive scores measured in early childhood among males. Specifically, an inverse multivariate adjusted association was observed between DNA methylation levels and the PPVT cognitive scores for cg05342682 (β = −3.2, 95% CI: −6.2, −0.2), cg21856205 (β = 4.5, 95% CI: −8.7, −0.1), cg17330251 (β = −1.9, 95% CI: −3.6, −0.1) and cg01874867 (β = −2.0, 95% CI: −3.7, −0.2). No significant associations were observed for individual CpG methylation levels of this region and WRAVMA cognitive test scores in males, Table 4. Although prenatal mercury exposure was not associated with *PON1* methylation in females, mean cord blood methylation levels of the *PON1* DMR was associated with lower cognitive test scores for the PPVT in girls measured in early childhood (β = −2.6, 95% CI: −4.8, −0.4; *P* = 0.021) but not WRAVMA scores, Supplementary Table S2. For the CpG-by-CpG analyses methylation levels of cg13340705 in the *WBP11P1* gene were marginally associated with WRAVMA cognitive test scores in the entire sample (β = 5.6, 95% CI: −0.6, 11.8; *P* = 0.07), Supplementary Table S3. Despite previously reported associations between prenatal mercury exposure and cognitive performance within a large subset of the Project Viva cohort^8^, we did not observe evidence for an association between log_2_-tranformed RBC-Hg and PPVT cognitive scores (β = 1.2, 95% CI: −1.0, 3.4; *P* = 0.29) or with WRAVMA scores (β = 0.4, 95% CI: −1.3, 2.0; *P* = 0.66) in our smaller sample. Therefore, we were unable to estimate the mediated effects of exposure on cognitive performance in this subsample of the cohort.

**Table 4:** Adjusted associations for methylation levels of the 9-CpGs in the DMR of the *PON1* gene in cord-blood of males with cognitive test scores measured during early childhood. Estimated change in cognitive test scores per 10% increase in methylation of each CpG and mean methylation levels of the *PON1* region.

*PON1* CpG ID	PPVT score (N = 135)		WRAVMA total (N = 128)	
Cord Blood Methylation	β-Coefficient (95% CI)	*P*	β-Coefficient (95% CI)	*P*
cg07404485	−2.1 (−3.7, −0.2)	0.19	0.03 (−2.2, 2.2)	0.97
cg05342682	−3.2 (−6.2, −0.2)	0.03	−1.5 (−3.7, 0.6)	0.15
cg04155289	−0.5 (−2.9, 4.0)	0.75	−0.4 (−2.8, 1.9)	0.72
cg19678392	−2.2 (−4.6, 0.2)	0.07	−0.4 (−2.1, 1.3)	0.64
cg21856205	−4.4 (−8.7, −0.1)	0.04	−1.1 (−4.2, 2.0)	0.48
cg17330251	−1.9 (−3.6, −0.1)	0.03	−0.3 (−1.5, 0.9)	0.64
cg01874867	−2.0 (−3.7, −0.2)	0.02	−0.4 (−1.7, 0.8)	0.48
cg20119798	−2.1 (−5.0, 0.7)	0.13	−0.5 (−2.4, 1.5)	0.64
cg04871131	−2.9 (−9.0, 3.2)	0.35	−1.1 (−5.3, 3.2)	0.62
Mean DMR methylation	−2.6 (−5.6, 0.3)	0.07	−0.6 (−2.7, 1.4)	0.55

(PPVT = Peabody Picture Vocabulary Test; WRAVMA = Wide Range Assessment of Visual Motor Abilities).

**Figure 7:**
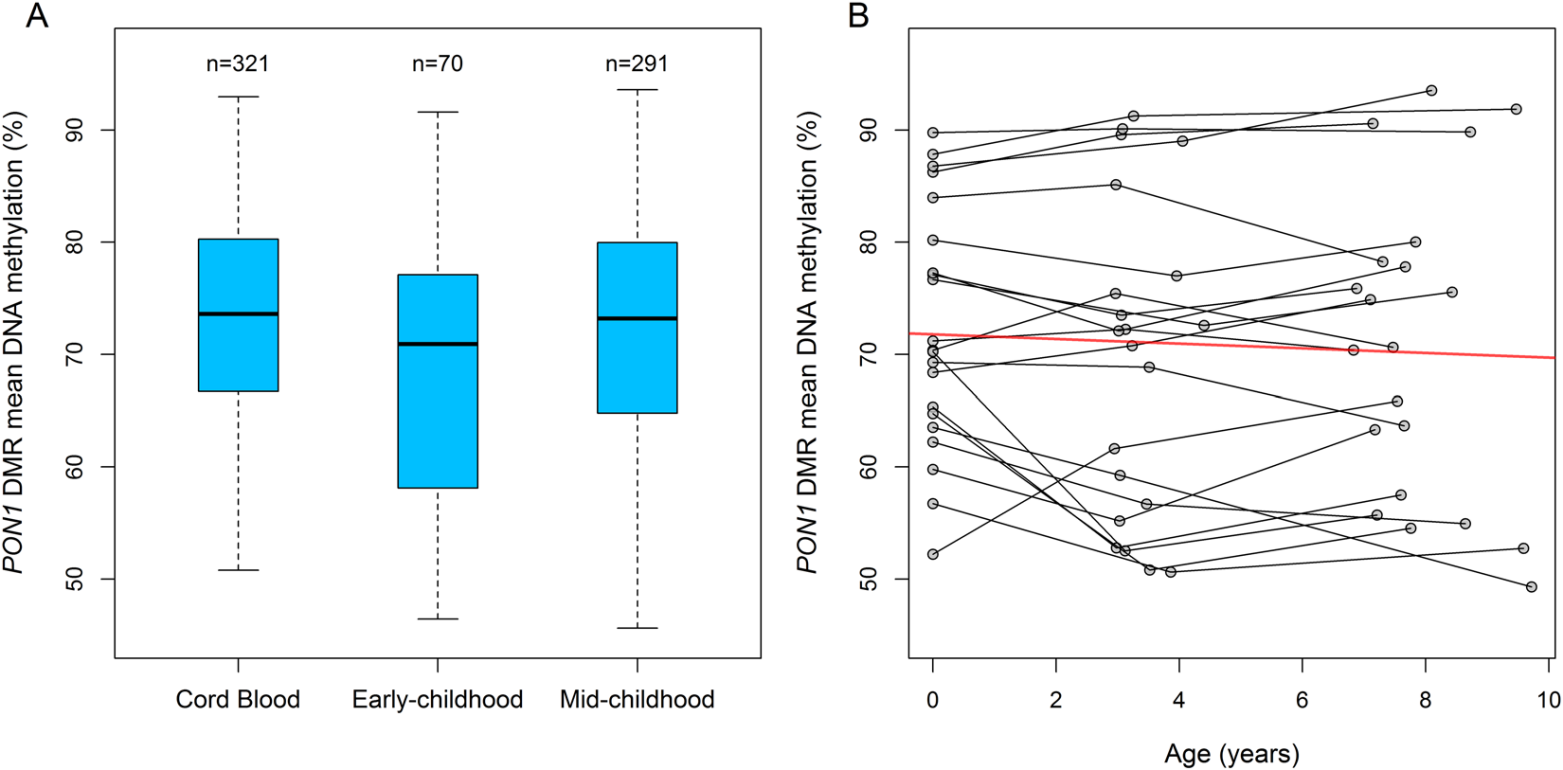
Mean methylation levels of the DMR in *PON1* measured at three time points: (**A**) Distribution of mean methylation levels among all individuals and (**B**) individual trajectories for participants that were repeatedly measured during the study period (n = 21) and fitted regression line (red).

Finally, we observed similar DNA methylation levels at the *PON1* DMR measured in cord blood and in early or mid-childhood (Fig. 7A). Among 21 participants with repeated DNA methylation measurements we observed no significant changes in methylation at the *PON1* DMR over time (β = −0.21%; 95% CI: −1.92, 0.78; *P* = 0.68), Fig. 7B.

## Discussion

In this study we observed DNA methylation changes of a genomic region of the *PON1* gene relative to prenatal mercury exposure in umbilical cord blood from males but not females, an association that persisted during early and mid-childhood. We also showed that higher DNA methylation levels of the *PON1* region are associated with lower cognitive test scores in early childhood for both sexes. Based on publicly available reference datasets we observed that cord blood DNA methylation levels of the *PON1* region is associated with decreased PON1 expression in cord blood. In individual CpG analyses, only one CpG of the *WBP11P1* gene was associated with prenatal mercury exposure, but this association did not persist in early or mid-childhood. In male-specific site-by-site analyses, a CpG of the *TOR4A* gene was hypermethylated relative to prenatal mercury exposure and this association persisted in mid-childhood. Taken together, these results suggest that moderate mercury exposure during pregnancy can lead to sex-specific functional epigenetic alterations that persist throughout childhood and are associated with cognitive performance.

The Paraoxonase 1 or arylesterase 1 gene (*PON1*) belongs to the serum paraoxonase family of enzymes previously shown to provide oxidative protection for high density lipoproteins and hypothesized to guard against atherosclerosis and metabolic syndrome^23, 24^. PON1 is synthesized in the liver and secreted into the plasma where it can metabolize toxic oxidized lipids^25^. The main substrates of PON1 are lactones, but promiscuous catalytic activity towards organophosphate triesters, arylesters, cyclic carbamates, glucuronides, oestrogen esters and thiolactones has also been documented^26^. Genetic polymorphisms in *PON1* and enzyme activity have been associated with adverse perinatal health outcomes such as decreased fetal growth, pre-term birth and reductions in head circumference^27, 28^. Genetic variants as well as low enzymatic activity of PON1 have been shown to contribute to the neurodevelopmental toxicity of organophosphorus (OP) pesticides in animal models and human studies^29, 30^. *PON1* protein has also been localized in brain regions of animal models as well as human tissue and experimentally shown to interact with other proteins in the brain that are essential for brain homeostasis^31^.

*In vitro* models and human studies have shown that MeHg exposure exerts an inhibitory effect on PON1 enzyme activity even after controlling for genetic variants including SNP_-108_ (rs705379), one of the major genetic determinants of PON1 expression^32–34^. The inhibitory mechanism of MeHg on PON1 has been hypothesized to be involved in the development of cardiovascular disease, potentially explaining epidemiological associations with MeHg^35^. Furthermore, PON1 enzyme activity is lower in neonates but relatively stable in adults exhibiting age-dependent developmental changes through at least 7 years of age. The developmental change in enzyme activity might create a sensitive window for toxic exposures during fetal development^25, 36, 37^. In fact, hypersensitivity to mercury exposure (acrodynia, or “pink disease”) among infants exposed to mercury has been attributed to genetic variation and subsequent enzyme activity of *PON1* ^38^. A large prospective epidemiological study showed that *PON1* genotype can modify the association of prenatal MeHg exposure and neurodevelopment, leading to stronger adverse effects in IQ deficits during childhood^39^. Interestingly, in a study of 896 Inuit adults, blood MeHg concentrations lowered PON1 enzyme activity independent of genetic variation, and there was no evidence of an interaction between MeHg and genetic variants of *PON1* ^32^. The lack of gene-environment interaction suggests a third control mechanism involved in the relationship between genetic variation, exposure and subsequent enzyme levels.

Although *PON1* genetic variants are better characterized, less is known about epigenetic control mechanisms. In a recent integrative genomic study, a single nucleotide polymorphism in *PON1* (SNP_-108_; rs705379) was shown to be strongly associated with DNA methylation subsequently mediating PON1 enzyme activity in newborns, an association that persisted to nine years of age^40^. In another study of obese adults, a similar inverse correlation between *PON1* methylation and catalytic activity of the enzyme was documented^41^. This is consistent with our observation in which increasing methylation levels at the *PON1* DMR decreased gene expression using data from an independent set of cord blood samples. These results highlight DNA methylation as a control mechanism involved in *PON1* expression, underscoring the importance of the male-specific *PON1* hypomethylation observed in our study relative to prenatal mercury exposure and subsequent association with cognitive test scores.

Sex-specific differences in DNA methylation for *PON1* are unknown for humans. However, in a case-control study of myocardial infarction, female specific hypermethylation of *PON1* was observed among cases compared to controls^42^. Moreover, animal studies have documented male-specific histone modifications along with increased expression of PON1 in male offspring exposed to a high fat diet *in utero* ^43^. This is consistent with our data in which male-specific hypomethylation of *PON1* was observed for prenatal mercury exposure, potentially leading to an increase in expression. The observed sex-specific epigenetic modification might help explain sex differences observed in mercury’s toxicity. For example, male-specific mercury associations have been documented in epidemiological studies for metabolic syndrome^44^, ADHD-related behavior, and neurodevelopment^45, 46^. An experimental study of mice prenatally exposed to mercury demonstrated that males have a higher expression of immune-related genes and miRNA levels compared to females, suggestive of potential sex-specific response during fetal development^47^.

DNA methylation of the *PON1* region could serve as a mediator of genotype and gene expression, explaining age-dependent changes in enzyme activity and modulation of mercury’s neurotoxicity, as shown for OP pesticide exposure. However, our study lacks information on genetic variants to estimate the effect of DNA methylation conditional on genotype. Therefore sex-specific epigenetic differences in the region could partially be driven by genetic variation. Nevertheless, increasing levels of DNA methylation in the *PON1* region was associated with a modest decrease in PPVT cognitive scores in both males and females, suggesting that epigenetic control of *PON1* could be implicated in cognitive development independent of prenatal mercury exposure or sex. As supported by the observed methylation-expression relationship in the BEAR birth cohort^22^ and previous studies^40^, DNA methylation regulates expression and enzymatic activity of PON1 providing a potential mechanism of mercury’s associated neurotoxicity. Furthermore *PON1* might also serve as an important target for other toxic effects of mercury exposure.

Although the growing interest in *PON1* has been so far focused in cardio-metabolic outcomes, there is evidence that suggests that *PON1* genotype and enzyme activity is associated with adult neurological diseases such as Parkinson and Alzheimer^48^. Furthermore, *PON1* genetic variants and enzymatic activity have been linked to autism spectrum disorder in cross-sectional studies^49, 50^. However, we did not measure DNA methylation in the brain as a target tissue a common limitation of prospective epidemiological studies.

The CpG found to be differentially methylated for the entire sample relative to prenatal mercury exposure is located in the TSS1500 region of the *WBP11P1* gene (cg13340705). The WW domain binding protein 11 pseudogene 1 (*WBP11P1*) has no known function and the association was not persistent in early or mid-childhood suggesting, that this modification is transient at birth. The single CpG (cg13416866) associated with prenatal mercury exposure in males in the Torsin Family 4, Member A gene (*TOR4A*) persisted into mid childhood but was not associated with cognitive scores. However, these individual loci have not been previously implicated in mercury toxicity, and the evidence for functional or phenotypic association with cognitive performance was limited.

Finally, in the three previous epigenome-wide studies of prenatal mercury exposure no overlaps between differentially methylated regions or CpGs have been documented. One study that included epigenome-wide analysis of placenta samples found that DNA methylation of the *EMID2* gene was associated with prenatal mercury exposure and neonate behavior. Interestingly both the *EMID2* and *PON1* gene are located in a region of chromosome 7 (q21–q31), experimentally shown to be sensitive to genomic imprinting using human-mouse monochromosomal hybrids^51^. Additionally, this region in *PON1* has been shown to have parent of origin specific methylation involved in the etiology of Silver-Russell syndrome, but *PON1* remains a disputed imprinted gene in humans^52^. Future studies should evaluate if prenatal mercury exposure targets other imprinted genes from this region. Heterogeneity of the DNA methylation targets across studies suggests that mercury’s associated epigenetic disruption might be time-sensitive during fetal development, emphasizing the importance of collecting both sensitive and time-specific biomarkers of exposure. Furthermore, mercury exposure levels in our cohort were relatively low but comparable to the general US population^53^. Therefore our results might not be generalizable to populations exposed to higher levels of mercury.

Our study has several limitations. First, in the absence of genotype information it is not possible to determine if genetic variants are responsible for the observed methylation differences. However, it has been shown that MeHg exposure inhibits PON1 activity even after controlling for the major known genetic variants, suggesting a dominant epigenetic control mechanism for the enzyme. While we observed associations between DNA methylation and gene expression these associations may not be generalizable to our cohort. Another important limitation of our study is the use of adult reference DNA methylation data to impute white blood cell distribution, as adult blood might not accurately capture leukocyte distribution in childhood. However, we used a cord blood reference panel to estimate cell proportions at birth, including nucleated red blood cells, and the persistence of epigenetic modifications were robust to white blood cell composition adjustment using different panels for cord blood and child blood. We also lack mercury exposure information during early and mid-childhood or any pesticide exposure information and therefore the relative contribution of postnatal exposures cannot be determined. The study was observational in nature and confounding cannot be ruled out.

The present study has many strengths that include the use of second trimester maternal red-blood cell mercury concentrations, an unbiased biomarker with an estimated half-life of 72 days^54^ and over 70% of the red-blood cell mercury is estimated to be methylmercury^55^. It is expected that this biomarker captured prenatal exposures occurring during critical windows of embryogenesis and fetal development. Although our sample size is moderate, this is the largest epigenome-wide study of prenatal mercury exposure conducted to date and the only one to prospectively test for persistence of epigenetic modifications at two time points during childhood. Our moderate sample size enabled us to evaluate sex-specific differences. Furthermore, this cohort has detailed anthropomorphic and demographic information that allowed us to control for many potential confounders and test for associations with cognitive test scores during early childhood. Detailed information on maternal diet also allowed us to control for fish intake, previously shown to be beneficial for children’s cognitive development, minimizing the possibility for negative confounding^8^. The analyses of regional and individual CpG sites methylation variability is also an important strength of the present study. Lastly, the potential for functional changes in DNA methylation with gene expression was evaluated in a separate set of cord blood samples further supporting our findings.

This study adds to the growing body of evidence that suggests that *PON1* serves as a potential target of toxic environmental exposures particularly during fetal development and childhood. Furthermore, DNA methylation of *PON1* could modulate the association between prenatal mercury exposure, cognitive development and other health outcomes in children. Epigenetic modifications leading to functional genomic changes could help explain heterogeneous findings observed for prenatal mercury exposure and cognitive development in different populations. This will also provide tools to design future targeted public health interventions.

## Methods

### Study Population

Mothers and children were participants in Project Viva, a prospective pre-birth cohort study conducted in Massachusetts, USA^56^. This cohort was recruited between 1999 and 2002 during their first prenatal visit at Atrius Harvard Vanguard Medical Associates, a large multispecialty group practice. Mothers provided written informed consent at study recruitment and at every follow-up visit. All study protocols were reviewed and carried out in accordance with guidelines approved by the human subjects committee of Harvard Pilgrim Health Care. Eligibility criteria included fluency in English, gestational age less than 22 weeks at the first prenatal visit, and singleton pregnancy. Additional details of the cohort have been published elsewhere^56^. Of the total 2,128 mother-infants pairs in the cohort, we measured prenatal mercury exposure from 1019. Of these, we included 321 with available measurements of cord blood DNA methylation. Persistence of the epigenetic modifications were evaluated in 75 children with available whole blood DNA methylation measurements from early childhood (2.9 to 4.9 years) and 291 children with blood samples from mid-childhood (6.7 to 10.5 years) as well as prenatal mercury concentrations. Early and mid-childhood participants were not restricted to participants with cord blood samples and therefore are not mutually inclusive. Of the 321 infants eligible for analyses with cord blood samples, 70 were included for early childhood analyses and 160 in the mid-childhood analyses. The number of participants sampled in both early and mid-childhood was 43.

### Red Blood Cell Mercury (RBC-Hg) and Fish Intake

Sample collection and exposure assessment for prenatal mercury exposure in this cohort has been previously described in detail^8^. Briefly, at the second trimester visit we obtained a maternal blood sample that was centrifuged to separate plasma from erythrocytes. Erythrocyte aliquots were stored at −70 °C and analyzed for total mercury by using the Direct Mercury Analyzer 80 (Milestone Inc., Monroe, Connecticut). Results are reported as mercury content in the original red blood cell sample. The detection limit was 0.5 ng/g of sample, and the percentage recovery for standards ranged from 90 to 110%. At mid-pregnancy, participants also completed a semi-quantitative food frequency questionnaire that we previously calibrated against erythrocyte levels of elongated n-3 fatty acids^57^. Participants self-reported on the consumption of fish with four questions: “canned tuna fish (3–4 oz.)”; “shrimp, lobster, scallops, clams (1 serving)”; “dark meat fish, e.g. mackerel, salmon, sardines, bluefish, swordfish (3–5 oz.)”; and “other fish, e.g. cod, haddock, halibut (3–5 oz.)”, during the preceding 3 months. Six frequency response options ranged from “never/less than 1 per month” to “1 or more servings per day.” We combined responses to estimate average total fish intake in servings per week.

### DNA Extraction and Sample Collection

Trained medical personnel obtained umbilical cord blood samples immediately upon delivery storing them in a dedicated refrigerator at 4 °C and transported to a central location within 24 hours of sample collection. Similarly, whole blood samples collected during early, and mid-childhood were stored at 4 °C and transported to the central storage location for sample processing. Subsequently, trained laboratory staff processed the samples on the same day of arrival, and DNA was extracted using the Qiagen Puregene Kit (Valencia, CA). Aliquots were then stored at −80 °C until analysis.

### DNA methylation Assessment and Quality Control

Buffy coat DNA was sodium bisulfite converted using the EZ DNA Methylation-Gold Kit (Zymo Research, Irvine, CA). Samples were allocated to plates using a two-stage algorithm by randomizing 12 samples to each chip and then randomly assigning eight chips to each of the 15 plates used to ensure balance by sex across chips and plates. Samples were shipped to Illumina Inc., and analyzed using the Infinium Human Methylation450 BeadChip (Illumina, San Diego, CA) following standard manufacturer’s protocols. The Human Methylation450 BeadChip measures DNA methylation at >485,000 CpG sites simultaneously at a single nucleotide resolution, covering 99% of the RefSeq genes.

We processed raw methylation image files using the *minfi* package in R^58^. Samples were excluded as potentially miss-labelled if they were mismatches on sex (n = 6), genotype (n = 6) or were deemed to be low in quality (n = 12). Technical replicates were also excluded from the analysis (n = 40). Correlation coefficients for individual probes among all technical replicates ranged from 0.98 to 1. We excluded individual probes if they had non-significant detection *P*-values (*P* > 0.05) for more than 1% of the samples. Additionally, non-CpG probes (i.e. rs and ch), probes in X and Y chromosomes, SNP-associated probes at either the single base extension or within the target region were removed for SNPs that have a minor-allele frequency of >5%. Previously identified non-specific and cross-reactive probes within the array along with polymorphic CpG loci were also excluded from the analysis^59^. After quality control on the probes, the total number of autosomal CpGs left for analysis was 384,349 loci for all the cord blood samples. Background correction and dye-bias equalization was performed via the normal-exponential out-of-band (*noob*) correction method^60^. Finally, a β-mixture quantile intra sample normalization procedure (BMIQ) was applied to the resulting data to reduce the potential bias that can arise from type2 probes^59^. For each CpG site, methylation is reported as average β-value = M/(M + U + ε), where M and U represent the average fluorescence intensity from the probe corresponding to the methylated and unmethylated target CpG and ε = 100 is a small quantity to protect against division by zero. Thus, the average β-value is an interval scaled quantity between zero and one interpreted as the fraction of DNA molecules whose target CpG is methylated.

We used ComBat^61^ to correct for Batch effects from plate and other potential sources of technical variability in methylation measurements. We visually inspected the effectiveness of adjustment for batch using the four main principal components before and after batch adjustment. Strip plots of control probes were visually examined for bisulfite conversion and specificity. Density plots for the β-values were examined across samples at each normalization step. Methylation values on the β-scale were logit transform to *M*-values as previously described to be more appropriate for differential analysis of DNA methylation^62^. All tables and results are presented on the β-value scale to ease interpretability.

### Cognitive Outcomes in Early Childhood

At the early childhood visit, trained research personnel administered the Peabody Picture Vocabulary Test (PPVT) and the Wide Range Assessment of Visual Motor Abilities (WRAVMA) cognitive tests. The PPVT evaluates receptive vocabulary for children age 2 or older based on a national reference sample and it is strongly correlated (*r* > 0.90) with verbal and full-scale intelligence quotient of the Wechsler Intelligence Scale for Children-III^63^. The Wide Range Assessment of Visual Motor Abilities (WRAVMA) evaluates domains of visual motor development and is moderately correlated with intelligence quotient (*r*~0.60)^64^.

## Statistical Analysis

We calculated means and standard deviations (SD) or sample size and percentage for all covariates to describe the study population during the three time points: birth, early childhood (2.9 to 4.9 years) and mid-childhood (6.7 to 10.5 years). White blood cell composition was estimated from DNA methylation measurements using the Houseman projection method^65^ from isolated cell types. To estimate cell types composition in cord blood we used a reference panel of nucleated cells isolated from cord blood (leukocytes and nucleated red blood cells)^66^ and an adult leukocyte reference panel for blood samples collected in early or mid-childhood as implemented in *minfi* ^58, 67^. We conducted a principal component analysis in the resulting 384,349 CpGs across all cord blood samples and used univariate linear regression models to explore associations among each of the top 30-principal components with phenotypic and technical covariates using the *EnMix* Bioconductor package of R^68^. All analyses were carried out using the R statistical package, version 3.2.3 (www.r-project.org/).

### Differentially Methylated Regional Analyses

We examined the association of prenatal mercury exposure with differentially methylated regions (DMRs) in cord blood using the R Bioconductor package DMRcate^69^. This regional analysis method was chosen over other available methods because it has an agnostic approach to both genomic annotation and direction of effect estimates. Briefly, this package first fits individual linear regression models using *limma* for each CpG and subsequently applies a Gaussian kernel smoothing function to test statistics grouping significant probes based on a maximum distance of λ = 1,000 base pairs. Significance testing among DMRs are adjusted for multiple comparisons using an FDR < 0.05. Regional analyses were performed in cord blood for all of the samples and also stratified by sex while adjusting for child gestational age at delivery, sex (if not stratified), and estimated nucleated cell types in cord blood (CD8^+^, CD4^+^, Natural Killer cells, Monocytes, B-cells and nucleated red blood cells). As well as maternal age, race/ethnicity, mean weekly fish intake during pregnancy, pre-pregnancy BMI, smoking during pregnancy, parity and college education.

Among significant DMRs found relative to log_2_-transformed mercury exposure we used robust linear models to estimate the association of each CpG in the region as well as mean methylation levels of all CpG sites in the DMR relative to maternal RBC-Hg concentrations adjusting for potential confounders. Persistence of the epigenetic changes was evaluated in early and mid-childhood blood DNA using similar robust linear models. Multivariate linear models were adjusted for the same covariates as the cord blood models with the exceptions that child race was used instead of maternal race, white blood cell composition was estimated from whole blood DNA methylation using an adult reference panel, and models were further adjusted by age of the child in days at the time of the blood draw. Associations were considered to be persistent with *P* < 0.05.

### CpG-by-CpG Analyses

We evaluated methylation differences at individual CpG sites in cord blood relative to maternal RBC-Hg concentrations using robust linear regression with heteroskedasticity-consistent estimators to model the methylation levels of each individual CpG on the *M*-value scale as the dependent variable and log_2_-transformed prenatal mercury exposure as the main predictor while adjusting for the same covariates as in the regional analyses. CpG-by-CpG analyses were further stratified by infant sex to evaluate sex-specific epigenetic disruption at individual sites. The genomic inflation factor (λ) for the overall analysis was 0.99 indicating of no major systemic biases. Statistical significance for the CpG-by-CpG analyses was evaluated using a Bonferroni adjusted level of significance (*P* < 1.3 × 10^−7^). Although the epigenome-wide analysis was performed on the *M*-value scale, all estimates are reported as percent change in methylation along with the corresponding *P*-values from robust linear regression models performed on the β-value scale, for ease of interpretability.

We carried forward individual loci with a *P* < 1.3 × 10^−7^ in cord blood analyses to subsequent analyses for blood samples collected during early and mid-childhood to evaluate the persistence of associations using multivariate robust linear regression models adjusting for covariates. We considered *P* < 0.05 as indicating the persistence of epigenetic alterations in early or mid-childhood for the CpG-by-CpG analyses.

### DNA Methylation and Gene Expression

To evaluate if the epigenetic alterations observed relative to prenatal mercury exposure are related to functional genomic changes in cord blood (i.e. expression), we used two publicly available datasets from an independent birth cohort found in the Gene Expression Omnibus (GEO) repository. Specifically, we identified DNA methylation data from 38 cord blood samples that used the Illumina Human Methylation450 array (GSE62924) and paired them with gene expression profiles from the same subjects (GSE48355) measured using the Affymetrix Human Gene 2.0 ST array. The study design, methods, sample collection and processing for this birth cohort have been previously described^22^.

We estimated correlation coefficients between gene expression and DNA methylation at CpGs within the DMR and at individual sites observed to be differentially methylated in cord blood relative to prenatal mercury exposure in our data. For the DMR found to be associated with prenatal mercury exposure, correlation coefficients between expression and mean methylation levels of the region as well as at individual CpGs within the region were tested. Correlation coefficients along with 95% Confidence Intervals (95% CIs) were used to estimate the association.

### Cord Blood DNA Methylation and Cognitive Performance

Linear regression models were used to estimate the association between differentially methylated DMRs or CpGs found in cord blood and cognitive test scores (PPVT and WRAVMA). Models were adjusted for maternal education at study enrollment, maternal PPVT scores, self-reported alcohol use during pregnancy, fetal growth (sex-specific z-score of birth weight/gestational age), mean weekly fish intake during pregnancy, child age in days at the time of testing, sex (if not stratified), child race, parity and maternal smoking during pregnancy. Estimates are reported for every 10% increase in methylation levels along with 95% CIs.

## Electronic supplementary material

Supplemental Material
