# Supplementary material for: Persistent DNA methylation changes associated with prenatal mercury exposure and cognitive performance during childhood
Source: Sci Rep. 2017 Mar 21;7:288. doi: 10.1038/s41598-017-00384-5 (PMC5428306; doi:10.1038/s41598-017-00384-5)

**Supplemental Material**

**Persistent DNA methylation changes associated with prenatal mercury exposure and cognitive performance during childhood**

Andres Cardenas, Sheryl L. Rifas-Shiman, Golareh Agha, Marie-France Hivert, Augusto A. Litonjua, Dawn Demeo, Xihong Lin, Chitra J. Amarasiriwardena, Emily Oken, Matthew W. Gillman, Andrea A. Baccarelli

**Table S1.** Correlation coefficients between individual CpG methylation levels and gene expression among loci observed to be differentially methylated in the CpG-by-CpG analysis relative to prenatal mercury exposure. DNA Methylation and gene expression data from an independent set of cord blood samples (n=38).

| **CpG ID** |  | **Gene** | **CHR** | **Relation to Island** | **Enhancer** |  | ***Correlation Coefficient* ρ (95% CI)** | ***P*** |
| --- | --- | --- | --- | --- | --- | --- | --- | --- |
| cg13340705 |  | *WBP11P1* | 18 | ---- | ---- |  | 0.07  (-0.25, 0.38) | 0.65 |
| **EWAS stratified by sex: Males (N=160)** | | | | | |  |  | |
| cg13416866 |  | *TOR4A* | 9 | Island | ---- |  | 0.17  (-0.15, 0.47) | 0.30 |

**Table S2.** Adjusted associations for methylation levels of the 9-CpGs in the DMR of the *PON1* gene in cord-blood of females with cognitive test scores measured during early childhood. Estimated change in cognitive scores per 10% increase in methylation of each CpG and mean methylation levels of the region.

| **CpG ID** | **PPVT Score (N=147)** | | **WRAVMA total (N=149)** | |
| --- | --- | --- | --- | --- |
| **Cord Blood Methylation** | ^ϯ^**β-Coefficient (95% CI)** | ***P*** | ^ϯ^**β-Coefficient (95% CI)** | ***P*** |
| cg07404485 | -2.8 (-5.2, -0.5) | 0.018 | -1.2 (-3.2, 0.7) | 0.21 |
| cg05342682 | -2.6 (-5.0, -0.3) | 0.025 | -1.4 (-3.3, 0.5) | 0.16 |
| cg04155289 | -3.6 (-6.6, -0.6) | 0.018 | -2.4 (-4.9, 0.1) | 0.05 |
| cg19678392 | -2.1 (-3.9, -0.4) | 0.018 | -1.2 (-2.6, 0.3) | 0.11 |
| cg21856205 | -4.2 (-7.4, -1.0) | 0.010 | -2.6 (-5.3, 0.1) | 0.05 |
| cg17330251 | -1.4 (-2.7, -1.0) | 0.034 | -1.1 (-2.1, 0.1) | 0.05 |
| cg01874867 | -1.5 (-2.8, -0.2) | 0.026 | -0.9 (-2.0, 0.2) | 0.10 |
| cg20119798 | -2.1 (-4.2, 0.01) | 0.05 | -1.1 (-2.9, 0.6) | 0.21 |
| cg04871131 | -3.7 (-8.8, 1.4) | 0.16 | -2.2 (-6.4, 2.0) | 0.30 |
| Mean DMR methylation | -2.6 (-4.8, -0.4) | 0.021 | -1.6 (-3.4, 0.3) | 0.09 |

(PPVT= Peabody Picture Vocabulary Test; WRAVMA = Wide Range Assessment of Visual Motor Abilities)

**Table S3.** Adjusted association of methylation levels in cord blood CpGs most strongly associated with maternal prenatal mercury concentrations (*P*<1.3x10^-7^) with cognitive test scores measured during early childhood (PPVT and WRAVMA)

| **EWAS: overall Population** | | | |  | **PPVT Score (N=282)** | |  | **WRAVMA total (N=275)** | |
| --- | --- | --- | --- | --- | --- | --- | --- | --- | --- |
| **CpG ID** |  | **Gene** | **CHR** |  | ^ϯ^**β-Coefficient**  **(95% CI)** | ***P*** |  | ^ϯ^**β-Coefficient**  **(95% CI)** | ***P*** |
| cg13340705 |  | *WBP11P1* | 18 |  | -2.9  (-11.7, 5.7) | 0.50 |  | 5.6  (-0.6, 11.8) | 0.07 |
| **EWAS stratified by sex: Males** | | | |  | **(N=135)** | |  | **(N=128)** | |
| cg13416866 |  | *TOR4A* | 9 |  | 4.5  (-1.8, 10.8) | 0.16 |  | 0.5  (-3.9, 4.9) | 0.83 |

^ϯ^Change in cognitive scores per 10% increase in methylation (PPVT= Peabody Picture Vocabulary Test; WRAVMA = Wide Range Assessment of Visual Motor Abilities; CHR = Chromosome)

**Figure S1.** Frequency histogram of the distribution of maternal red blood cell mercury (RBC-Hg) concentrations measured during the second trimester of pregnancy **A)** untransformed frequency distribution and **B)** log_2_-transformed frequency distribution

**
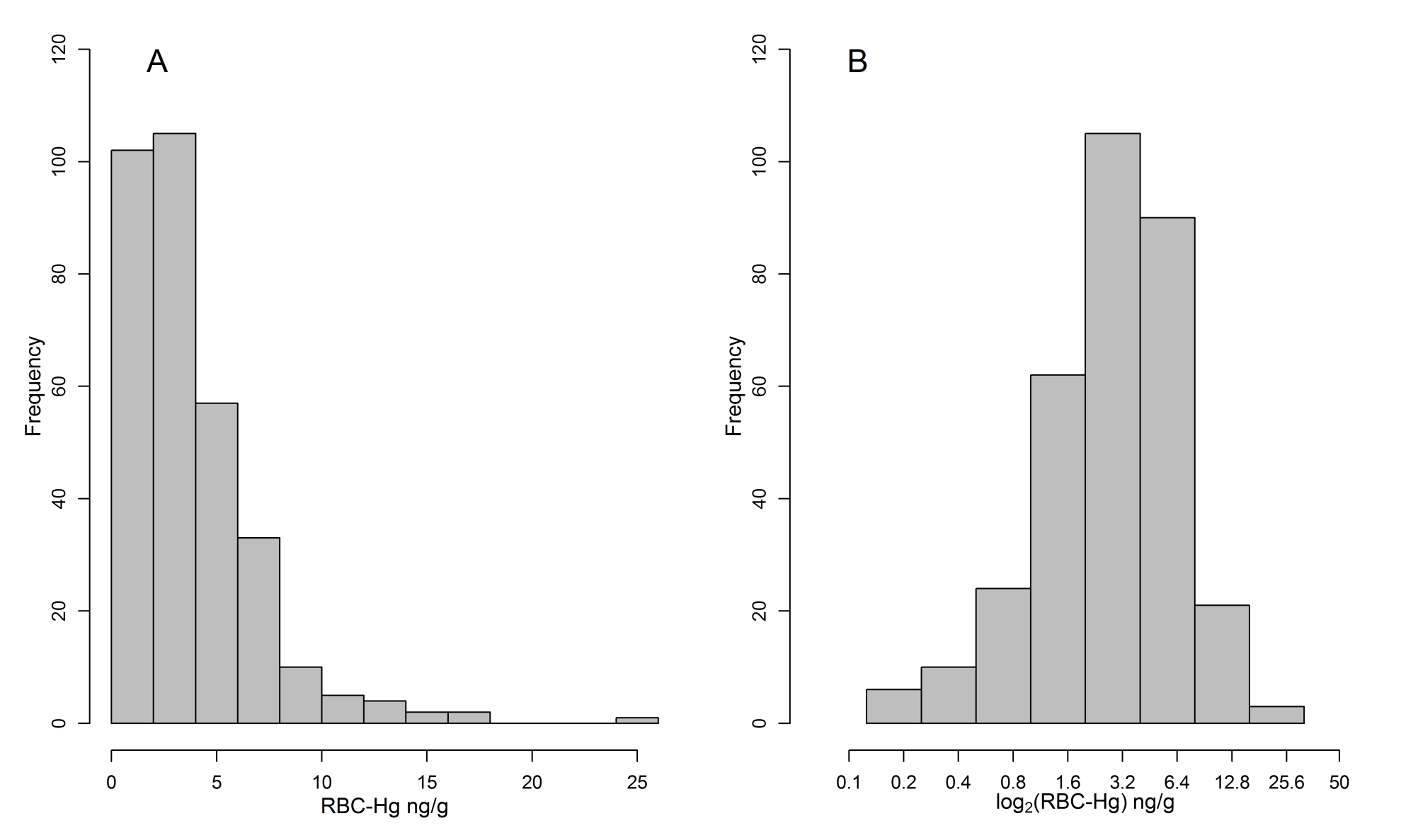
**

**Figure S2.** Annotated genomic tracks of the DMR within the *PON1* gene: expression array coverage (purple), common SNPs, human mRNAs (black block), CpG island location (green block) and individual CpGs annotated along the region.


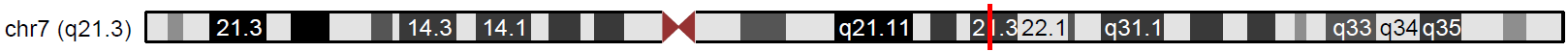


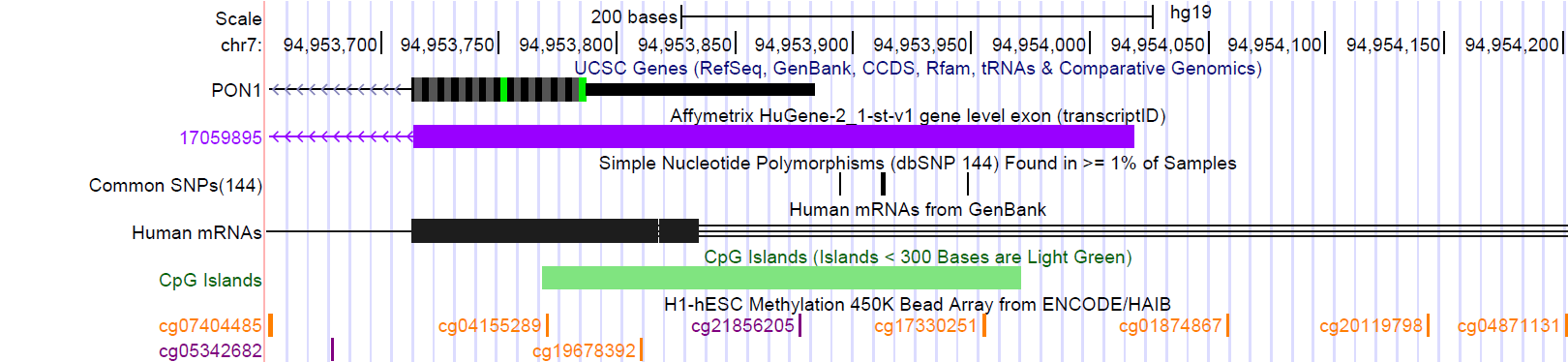

Supplement: Supplementary file 1 — Supplemental Material [file 41598_2017_384_MOESM1_ESM.doc]
